# Template-Directed Replication and Chiral Resolution during Wet–Dry Cycling in Hydrothermal Pools

**DOI:** 10.3390/life13081749

**Published:** 2023-08-15

**Authors:** David Ross, David Deamer

**Affiliations:** 1 SRI International, Menlo Park, CA 94025, USA; 2 Department of Biomolecular Engineering, University of California, Santa Cruz, CA 95064, USA

**Keywords:** non-enzymatic polymerization of nucleotides, assembly of duplex polymers, replication of homochiral structures

## Abstract

The commonly supposed template-based format for RNA self-replication requires both duplex assembly and disassembly. This requisite binary provision presents a challenge to the development of a serviceable self-replication model since chemical reactions are thermochemically unidirectional. We submit that a solution to this problem lies in volcanic landmasses that engage in continuous cycles of wetting and drying and thus uniquely provide the twofold state required for self-replication. Moreover, they offer conditions that initiate chain branching, and thus furnish a path to autocatalytic self-replication. The foundations of this dual thermochemical landscape arise from the broad differences in the properties of the bulk water phase on the one hand, and the air/water interfacial regions that emerge in the evaporative stages on the other. With this reaction system as a basis and employing recognized thermochemical and kinetic parameters, we present simulations displaying the spontaneous and autocatalyzed conversion of racemic and unactivated RNA monomers to necessarily homochiral duplex structures over characteristic periods of years.

## 1. Introduction

Although most of the research related to the origin of life is performed in the laboratory, life did not begin at a bench with pure reagents, controlled temperatures, and buffered media [1]. Two widely considered views of its origins suggest that life might have begun at hydrothermal vents in salty seawater [2] or in hydrothermal freshwater sites associated with subaerial volcanic land masses, resembling Hawaii and Iceland today [3,4]. While an extensive literature has developed supporting the former with its focus on a primitive version of metabolism and CO_2_ reduction, some accounts have taken issue with that approach [5,6,7]. We present here an exploration of the second path, proposing that in hot spring conditions the essential polymerization reactions can be overall exergonic and thermochemically driven by cycles of wetting and drying. We present both theoretical considerations and laboratory experiments designed to test this approach.

### 1.1. Hydrothermal Conditions Associated with Volcanism and Prebiotic Analogue Sites

**Figure 1:**
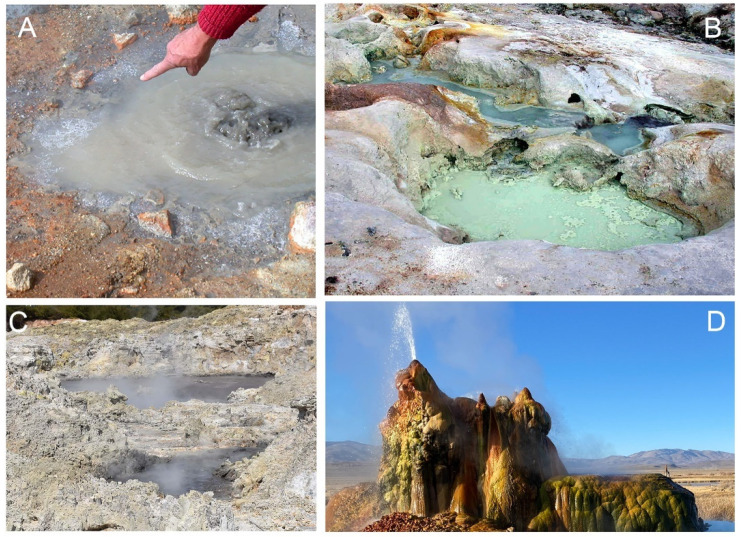
Examples of prebiotic analogue conditions. (**A**) A boiling, hydrothermal pool on Mount Mutnovsky, an active volcano south of Petropavlovsk, Kamchatka, Russia. The author is pointing at a membranous froth produced by myristic acid added to the pool [4]. Photo credit: Tony Hoffman). (**B**) Hydrothermal water in Bumpass Hell on Mount Lassen, California (photograph by author). (**C**) Hydrothermal pools in Hell’s Gate, near Rotorua, New Zealand (Photograph by author). (**D**) Fly Geyser in the Black Rock playa of northern Nevada (credit: Bruce Damer).

To understand the complexity of hydrothermal conditions, it is useful to consider prebiotic analogue sites. Several of these are illustrated in Figure 1, including Mount Mutnovsky in Kamchatka, Russia, Bumpass Hell on Mount Lassen in California, Hell’s Gate near Rotorua, New Zealand, and Fly Geyser in the Black Rock playa of northern Nevada. A laboratory scientist visiting such sites will be surprised by their apparent complexity, but some general properties of hydrothermal sites can be listed:

- The water is distilled from salty seawater by evaporation and supplied to the sites by cycles of precipitation;
- The pools tend to be acidic due to the presence of SO_2_, which is hydrolyzed to sulfurous acid and in turn oxidized to the stronger sulfuric acid;
- The temperature of the pool water is typically in the range of 90 °C, but varies depending on the altitude of the site;
- The ionic solutes present in hydrothermal fields are provided by the minerals that line the pools. Most of the minerals are in the form of basaltic lava and volcanic ash, as well as clays that are produced when basalt is exposed to elevated temperatures and acidic conditions;
- Unlike hydrothermal vents, hydrothermal pools undergo continuous cycles of wetting and drying. If they are small enough, entire pools can evaporate in days between periodic precipitation, but shorter wet–dry cycles occur around the borders of larger pools. Hot spring splashing and geyser activity also produce short wet–dry cycles measured in minutes;
- We assume here that mononucleotides were present in prebiotic hot spring pools. Recent progress has been made in possible synthetic reactions in which mononucleotides are products [8,9].

Wet–dry cycles have several important properties supporting a potential origins path. Evaporation concentrates potential reactants as films on mineral surfaces, and amphiphilic components will readily encapsulate polymeric products of condensation reactions in microscopic membrane-bounded compartments. Given a source of monomers and amphiphiles, these characteristics offer a one-pot assembly of protocells driven by the medium-sensitive qualities of condensation reactions and the inherent fluctuating dynamics of wet–dry cycles.

### 1.2. Origins and Cooperative Reaction Environments

From a paired Occam’s Razor and RNA-First perspective, the challenges faced in shaping a path to life’s beginning were severe. A successful reaction system required an energy landscape that would ultimately provide exponential growth, and it thus required products that were both self-replicating and potentially catalytic. The task was confronted further by a starting reactant pool of unactivated racemic starting materials. In accord with recent observations by Bare and Joyce [10], we propose that a successful process could have emerged from a primitive version of the present-day polymerase chain reaction, including template-directed synthesis, double-stranded polymer unpairing, and a chain-branching sequence, all without the benefit of triphosphate activation and the highly precise control of enzyme-catalyzed reactions.

On this basis, life’s origins required two reaction settings that employed two different and opposing reaction paths. The first would utilize highly concentrated solutions in which molecular crowding and the shifted properties of an aqueous medium in interfacial regions would overcome the inherent antientropic qualities of condensation reactions and yield duplex structures that were necessarily homochiral [11,12,13]. The second would operate at highly dilute conditions and incorporate Le Chatelier’s principle to unpair the duplexes to yield homochiral oligomers, which then return to the highly concentrated condition and engage in template-directed replication, chain branching, and autocatalytic growth.

As we shall illustrate here, the varied collection of interfacial and bulk medium regions within prebiotic wet–dry cycling hydrothermal pools uniquely provided both of those highly contrasting but necessary conditions. We address the matter with an approach derived from our recent description of spontaneous deracemization leading to the formation of populations of homo-*l* and homo-*d* duplexes from unactivated and racemic RNA monomers [13]. Our approach applied both the upending of the Gibbs energy for phosphate/nucleoside ester formation to an exergonic process at the air–water interface [14] and the fact that duplexes are inherently homochiral. We then employed the uncommonly rapid rate and cumulative exergonicity of duplex formation [15] to the pairing of the homochiral complement pairs that, while statistically at very low concentrations, must be present in aqueous solutions of RNA oligomers at equilibrium. Computational simulations integrating those kinetic and thermochemical elements with the appropriate statistical factors then revealed a path to spontaneous deracemization and the full conversion of the starting racemic monomers to product duplexes that were necessarily homochiral. With those observations as a foundation, we now address the essential dual role of wet–dry cycling.

## 2. Results

### 2.1. Replication Scheme

Our goal here is to integrate a set of conditions that support a reaction sequence employing racemic and unactivated RNA monomers, resulting in exergonic autocatalytic template-assisted replication in a prebiotic setting. The available tools include the shift from endergonic to exergonic ribose/phosphate linkages at the air–water interface [11,12,14]. Eigen’s kinetic and thermochemical observations [15], and the hydrophobic effect that governs molecular assembly [16]. The most significant driver is a branching chain process in which each fashioned component itself becomes a catalytic template that promotes exponential replicative growth. Branching chains are commonly recognized in combustion processes where the exponential production of a catalytic component leads to rapidly mounting reaction rates [17]. Uranium fission is a familiar example of a branching kinetics process wherein the neutron production must be controlled by a moderator to avoid an explosion. A branching polymerization reaction in an early earth scenario yields a less dramatic product, but within the context of an origins scheme, the result—primitive life—is no less spectacular.

**Scheme 1:**
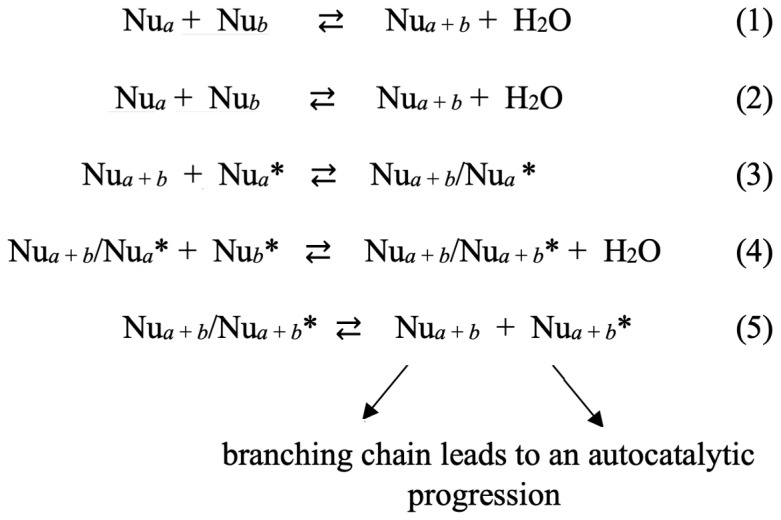
Series of reactions leading to a branching chain and autocatalytic progression.

Our proposed path, adapted from our recent account [13] and employing a branch point, is depicted in Scheme 1. It is initiated in Equation (1) for *a* = *b* = 1 and advances *iteratively* to a series of RNA oligomers. Equation (2) is a subset of (1), involving solely homochiral oligomers that are identified in the scheme with underscoring, and Equation (3) portrays partial duplex assembly with an asterisk identifying the corresponding oligomer complement. Equation (4) depicts a reaction with a second complementary fragment to yield the full duplex, and the equivalence of Nu*_a_*_+*b*_ and Nu*_a_*_+b_* as templates in Equation (5) provides the operational heart of the scheme: chain branching and an autocatalytic path. Notably, all of the steps in the sequence are recognized as reversible and the homochiral oligomers are thus readily backfilled as they are expended, leading in the end to full conversion. 

The unpairing in Equation (5) is thermally driven in present-day PCR technology and requires meticulously controlled temperature programming [18]. In a prebiotic setting, however, duplex separation would be achieved by dilution at the elevated temperatures of hot springs, as can be recognized with reference to Equation (6) which was developed from the predictive nearest neighbor models for duplex formation [13]. The

ΔG^o^_*a-* or *b*mer mating_ = ΔG^o^_bimolecular/symmetry_ + (*a* or *b*)ΔG^o^_stacking_ (6)

expression includes a term representing penalties for association (ΔG^o^_bimolecular/symmetry_) that sum to about 5 kcal/mol and a penalty-opposing series of ten sequence-dependent stabilizing parameters (ΔG^o^_stacking_) that can override the association penalties and lead to an overall exergonic assembly. For example, a 7/7 duplex has a ΔG^o^_*a-* or *b*mer mating_ ≈ −10 kcal/mol. Dilutions down to about 5 nM, however, will afford an unpairing of ~90%. Greater dilutions yield still larger unpairing levels and in the end provide a population comprising homochiral oligomers with a range of lengths. 

### 2.2. Kinetic Simulations: Computational Testing of the Process 

We examined Scheme 1 in kinetic simulations at 25 °C, employing the Kintecus modeling package [19]. Presuming an overall flat nucleotide distribution for each value of *a* and *b*, we directed Kintecus to engage the four starting racemic and unactivated RNA monomers through all combinations of *n*mer production up to *n* = 50. The condensation and unzipping steps were conducted, respectively, in the interfacial and bulk medium regions. Thermochemical direction was provided by Equation (6), while the kinetic factors were developed from the notion that the homochiral fraction for each of the 4*^n^* sequences that develop for each value of *n* is 2/8*^n^*. Then,

rate _duplex formation_ = (0.5 k_assoc_) (c*_a-_*_or *b*mer_) (c_template_/16^*a*^
^or *b*^) (7)

where k_assoc_ = 10^7^ M^−1^ s^−1^. For a 7mer at 20 mM, the homochiral components will be at steady-state concentrations of no more than about 20 nM. The near-encounter value of k_assoc_ nonetheless yields a half-life for duplex formation of about 3 s. Even at nanomolar concentrations, duplex formation in Equations (3) and (4) is one of the most rapid reactions in the sequence. In the end, both the comprehensive reversibility in Scheme 1 and the −10 kcal/mol stability of a 7/7 duplex lead to essentially a full conversion to duplex structures in the interfacial region. 

**Figure 2:**
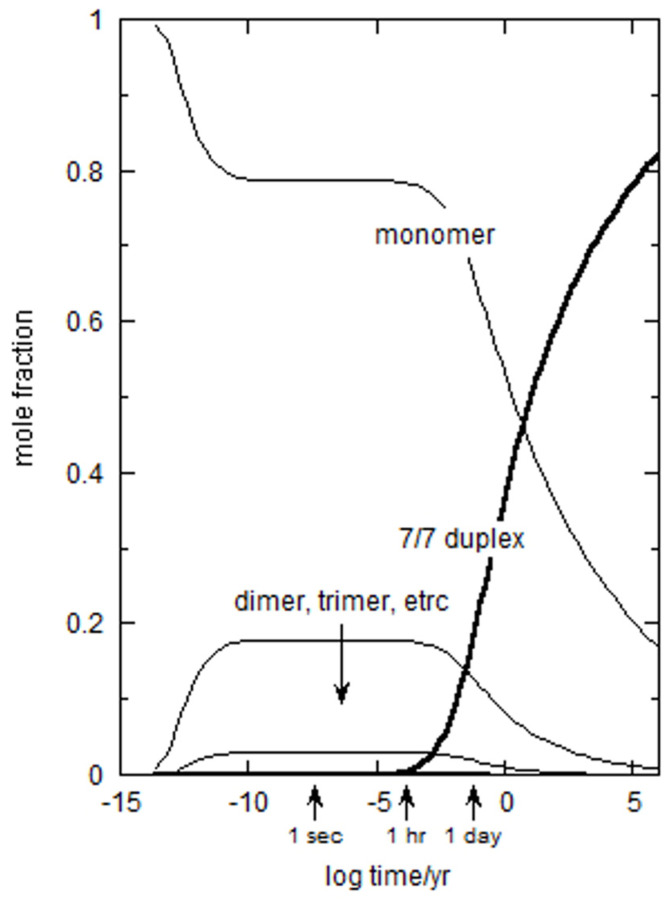
The production a 7/7 duplex via Scheme 1. The starting solution contained equimolar quantities of unactivated and racemic versions of the four RNA monomers at an overall concentration of 2.3 × 10^−2^ M.

With the details of this approach recently described in greater depth [13], a summary of the thermochemical and kinetic values used in the simulations is provided in Table 1, and the results of simulations of 7-template/3 + 4 and 7-template/4 + 3 → 7/7 duplexes, are presented in Figure 2.

The starting solution contained equimolar quantities of unactivated and racemic versions of the four RNA monomers at an overall concentration of 2.3 × 10^−2^ M. The figure exhibits the initial formation of a family of oligomers over just fractions of a msec. The subsequent emergence of the 7/7 duplex occurs over periods of mere hours, consistent with the very high rates of both esterification and duplex formation in the interfacial region. The sigmoidal form of the profile reveals the autocatalytic nature of their production, propagating exponentially over a few tens of years to become the dominant components of the solution. The profile represents equal quantities of the product homo-*d* and homo-*l* duplexes present in all conceivable sequences, but it is to be noted that this distribution is an artifact of the modeling process, wherein an average value of ΔG^o^_stacking_ has been employed. The ΔG^o^_stacking_ parameters (listed in Table 4 have values ranging from −0.93 kcal/mol for 5′AA3′/3′UU5′ to a substantial −3.42 kcal/mol for 5′GC3′/3′CG5′ [21]. In accord with earlier reflections related to tRNA sequences [22] sequence formation in nature will be governed initially by thermodynamic factors, but as complexity increases in later generations we can expect functionality and performance to guide structural evolution. 

### 2.3. Proposed Experimental Tests of the Process

There is now a substantial weight of evidence that wet–dry cycles at elevated temperatures can drive the polymerization of unactivated mononucleotides [8,9,23,24]. Single mononucleotides would be expected to form single-stranded products, and this was confirmed using nanopore analysis [23]. In some experiments with binary mixtures of mononucleotides capable of Watson–Crick base pairing, it was reported that the products exhibited the hyperchromicity characteristic of thermal unzipping, as though duplex strands had been synthesized. However, the mononucleotides in this experiment were homochiral compounds composed of D-ribose. A straightforward prediction of the process described here is that duplex structures exhibiting hyperchromicity will also emerge when a racemic mixture of mononucleotides undergoes polymerization driven by wet–dry cycling.

## 3. Discussion

We will begin by summarizing the series of steps involved in the proposed pathway to exponential template-assisted replication. This list serves as a guide to experimental tests related to the prediction that homochiral polymers will emerge from non-activated, racemic nucleotides when they are exposed to simulations of wet–dry cycles occurring in hot spring conditions of the prebiotic Earth.

- It is assumed that mixtures of racemic mononucleotides were available on the early Earth [1,25];
- The monomers polymerize into linear, largely racemic strands in the dry-down phases of a series of wet–dry cycles. Small but statistically significant concentrations of *d*- and *l*-oligomers will reside in the population and are the central components in what is to follow;
- Impelled by the exergonic driver expressed in Equation (6), they undergo Watson–Crick pairing with their respective complements at near encounter rates;
- In the subsequent wet portion of the cycle the oligomer pairs separate, providing *d*- and *l*-oligomers;
- The succeeding dry-down phase is then primed for chain branching and exponential growth.

**Figure 3:**
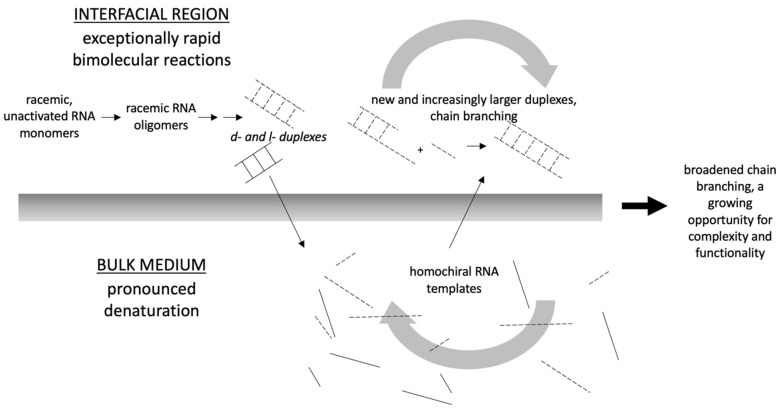
Schematic depiction of the development of chain branching and exponential self-replication resulting from cyclic pond evaporation and filling.

Through a succession of evaporation and hydration sequences and a deepening Gibbs energy well, a starting solution of racemic and unactivated RNA monomers is channeled into a branching chain progression, exponential growth, and in the end to duplexed structures with increasing levels of complexity and potential functionality. The overall process is sketched in Figure 3.

It is notable that the rate constant of 2.5 × 10^5^ M^−1^ s^−1^ for ester formation in the interfacial region [14] is consistent with the kinetic data for a large family of bimolecular reactions studied in films and microdroplets, with constants ranging over 10^2^–10^7^ M^−1^ s^−1^. They are uniformly many orders of magnitude greater than their respective bulk media values [26,27] while significantly there were no kinetic differences in interface/bulk medium rate comparisons for a series of first order reactions. The fact that interfacial water is organized into an ice-like, low-entropy structure [28] suggests that the structure itself could act as a matrix for assembly chemistry, a notion supported by the recent observations of McDermott et al. that the helical structure of DNA is stabilized by “crystallographic water molecules” in the minor DNA groove, leading to the suggestion of a chiral “spine of hydration” [29]. An interface-dependent mechanism was also invoked by [30], who reported a remarkable synthesis of extraordinarily long polymers when adenosine monophosphate in solution was exposed to a dispersion of graphite coated with anthraquinone. Gel electrophoresis revealed polymers up to 4000 nucleotides in length.

### 3.1. Depurination

There is one other degradative reaction that must be taken into account, the hydrolytic breakage of the N-glycoside bond between purine nucleotides and the ribose moiety of the polymer backbone. This process is continuous in aqueous solutions of purine mononucleotides like adenosine monophosphate and guanosine monophosphate, while pyrimidine mononucleotides are relatively stable. For instance, approximately 20 percent of an AMP solution undergoes depurination after eight 30-min wet–dry cycles at pH 2 and 85 °C [31]. However, in the presence of a lipid—lysophosphatidylcholine—depurination under the same conditions is reduced to ~1 percent. On a broader kinetics basis it emerges that the branching chain sequence described here leads to exponential increases in the rates of duplex formation that quickly overtake rates of depurination and suppress its significance. That dominance can be seen in a comparison of the depurination half-life of native DNA at 25° of about 9 years [32] with the ordinate value of the sigmoid in Figure 2 at the 1-day mark that corresponds to a doubling time of several days. 

### 3.2. Activation of Monomers Has Limited Value in the Prebiotic World

All life today utilizes chemically activated mononucleotides in the form of nucleoside triphosphates, for which enzymatically catalyzed polymerization is exergonic. Although imidazole esters have been established as laboratory models of activated mononucleotides [33,34], a plausible mechanism by which mononucleotides can be activated in prebiotic conditions has not been established. We emphasize that the process we have described here employs unactivated mononucleotides. The commonly presumed need for activation, variously justified through a range of factors [35,36], arises from the fact that the synthesis of phosphate/nucleoside esters is endergonic in bulk solution [37]. Activated monomers commonly employed in oligonucleotide studies are synthesized with imidazole-, polyphosphate-, and phosphoramidate-bearing substituents linked to the 5′- position; they are then highly reactive and ensure exergonic linking. Activation is effective in the laboratory but brings with it a number of issues. They include chain-suppressing cyclization and the competitive hydrolysis of activated nucleotides [36], sporadic production of 2′–5′ rather than 3′–5′phosphodiester linkages [38] and shorter rather than the requisite longer oligomer sequences [13]. Most significantly, there is at present little evidence supporting activation as a precondition in a prebiotic setting, and indeed the common notion that activators were supplied by prebiotic volcanism is difficult to defend. Volcanic eruption rates on early Earth were greater than present-day rates by a factor of about five [39] but the events were episodic. Only a sustained source of activated monomers could overcome the inexorable advance of exergonic hydrolytic decomposition [12].

Activation when applied to prebiotic template-directed RNA synthesis is particularly problematic. In contrast to laboratory studies, wherein base-coordinated monomers and templates are commonly employed, a prebiotic venue will necessarily make all four RNA monomers available for polymerization. Activation promotes esterification for *every* case, but attachment of the “wrong” monomer eliminates that template/primer contender from any further participation in replication [40]. A total of three out of four prospective sites for replication will be lost with each addition cycle, with the fraction growing to seven of eight in the case of racemic monomers. This path to degradation is exponential and will eliminate more than 98% of the potential growth sites within the first two or three cycles. The disqualified components, moreover, continue to consume activated monomers as they persist in the futile production of useless products. The results described here underscore the value of a branching chain sequence in a prebiotic environment in which degradative reactions represent a barrier to the assembly of the large and complex structures required for life’s origin. The data of Oivanen et al. [41] for example, reveal that the half-life of an *n*mer in the bulk aqueous medium at 90 °C ranges from ~300/(*n* − 1) hours at pH 7 down to ~1000/(*n* − 1) seconds at pH 2, a pace that would quickly overwhelm an unbranched polymerization. A branching chain sequence such as described here incorporates a kinetic leverage that will counter degradation and open a productive path to the complex and fragile molecular structures that led to life.

## Figures and Tables

**Table 1:** Values employed in the Scheme 1 simulations.

Items	Values	Source
Starting overall monomer concentration	2.3 × 10^−2^ M (solubility limit of ADM, 25 °C)	Wang, et al. (2010) [20]
ΔG^o^_1_, ΔG^o^_2_	−1.1 kcal/mol	Nam, et al. 2017 [14]
k_1_, k_2_	2.8 × 10^5^ M^−1^ s^−1^	Nam, et al. 2017 [14]
ΔG^o^_3_, ΔG^o^_4_, ΔG^o^_−5_	3/3, −1.3 kcal/mol 4/4, −3.4 kcal/mol 7/7, −9.7 kcal/mol ^a^	Equation (6), ΔG^o^ _bimolecular initiation/symmetry_ = 5 kcal/mol, ΔG^o^_stacking_ = −2.1 kcal/mol (average); Ross and Deamer 2022 [13]
k_3_, k_4_^a^_,_ k_−5_	functions of *a*, *b*	Equation (7), k_assoc_ = 10^7^ M^−1^ s^−1^; Eigen (1971) [15]

^a^ Linking of the second oligomer in Equation (5) includes both ester formation and stacking. For its Gibbs energy value, we have included only the stacking contribution and view the value as an upper limit. For k_5_, we presume the rate of ester formation to be incorporated within k_assoc_.

## Data Availability

Not applicable.
